# Consultation liaison psychiatry after COVID-19

**DOI:** 10.1192/j.eurpsy.2021.678

**Published:** 2021-08-13

**Authors:** M. Hernandez Alvarez

**Affiliations:** Psychiatry And Psychotherapy, Consultas Vithas La Milagrosa, Madrid, Spain

**Keywords:** psychotherapy, Organizational Consultancy, covid 19, Consultation Liaison Psytriatry

## Abstract

**Introduction:**

The paper will describe the experience as consultation-liaison psychiatrist during the Covid 19 Pandemic in a private hospital in Madrid, what we have learned and its implications given the considerable and increasing interest in European Consultation-Liaison research.

**Objectives:**

Following the request of one of the internal medicine department doctors the service was initially provided for patients admitted with the infection but very quickly included relatives and also the hospital staff.

**Methods:**

Patients were offered a telephone consultation that in most cases took place on a daily basis. Referrals where made by a doctor, some of them were locums due to the increasing demands of the service since patients from public hospitals were also admitted. Relatives were also referred by doctors and the frequency was more varied, depending on their needs. Members of the multidisciplinary team referred themselves..

**Results:**

Patients and their families felt that the telephone consultation was useful to them. The work with some members of the staff is ongoing and will continue given the toxic levels of stress that they had to face and the changes taking place at the institution at the time.

**Conclusions:**

The COVID-19 pandemic and the short and long term consequences that will follow will increase our understanding the breadth and depth of Consultation-Liaison Psychiatry and the broad perspective required for a comprehensive evaluation and treatment of patients. My experience as psychoanalytic psychotherapist and organizational consultant proved most helpful.

